# Impaired folate 1-carbon metabolism causes formate-preventable hydrocephalus in glycine decarboxylase–deficient mice

**DOI:** 10.1172/JCI132360

**Published:** 2020-02-04

**Authors:** Chloe Santos, Yun Jin Pai, M. Raasib Mahmood, Kit-Yi Leung, Dawn Savery, Simon N. Waddington, Andrew J. Copp, Nicholas D.E. Greene

**Affiliations:** 1 UCL Great Ormond Street Institute of Child Health and; 2 EGA Institute for Women’s Health, University College London, London, United Kingdom.; 3 MRC Antiviral Gene Therapy Research Unit, Faculty of Health Science, University of the Witswatersrand, Johannesburg, South Africa.

**Keywords:** Development, Neuroscience, Mouse models, Neurodevelopment, Neurological disorders

## Abstract

Ventriculomegaly and hydrocephalus are associated with loss of function of glycine decarboxylase (Gldc) in mice and in humans suffering from non-ketotic hyperglycinemia (NKH), a neurometabolic disorder characterized by accumulation of excess glycine. Here, we showed that ventriculomegaly in Gldc-deficient mice is preceded by stenosis of the Sylvian aqueduct and malformation or absence of the subcommissural organ and pineal gland. Gldc functions in the glycine cleavage system, a mitochondrial component of folate metabolism, whose malfunction results in accumulation of glycine and diminished supply of glycine-derived 1-carbon units to the folate cycle. We showed that inadequate 1-carbon supply, as opposed to excess glycine, is the cause of hydrocephalus associated with loss of function of the glycine cleavage system. Maternal supplementation with formate prevented both ventriculomegaly, as assessed at prenatal stages, and postnatal development of hydrocephalus in Gldc-deficient mice. Furthermore, ventriculomegaly was rescued by genetic ablation of 5,10-methylene tetrahydrofolate reductase (Mthfr), which results in retention of 1-carbon groups in the folate cycle at the expense of transfer to the methylation cycle. In conclusion, a defect in folate metabolism can lead to prenatal aqueduct stenosis and resultant hydrocephalus. These defects are preventable by maternal supplementation with formate, which acts as a 1-carbon donor.

## Introduction

Hydrocephalus results from abnormal cerebrospinal fluid (CSF) hydrodynamics (overproduction, diminished drainage, or impaired flow) leading to progressive enlargement of the cerebral ventricular system and subsequent pathology (1). Hydrocephalus can be acquired (following injury, infection, tumor formation, or trauma) or arise as a congenital condition, in isolation or associated with structural abnormalities such as Dandy-Walker or Chiari II malformations (2, 3). Although common (affecting 0.5–1 per 1,000 live births), the molecular pathophysiology of isolated congenital hydrocephalus is known in only a relatively small proportion of cases, owing to heterogeneity and multifactorial etiology (4–6).

A potential causal effect of impaired function of the glycine cleavage system (GCS) is highlighted by occurrence of hydrocephalus in association with non-ketotic hyperglycinemia (NKH), a life-limiting autosomal recessive neurometabolic disorder characterized by accumulation of glycine in body fluids and tissues (7, 8). NKH results from mutation of GCS-encoding genes, with the majority of patients carrying mutations in *GLDC* (glycine decarboxylase) (9, 10). Hydrocephalus arises in approximately 8% of NKH patients and enlarged ventricles are commonly found on imaging (15 of 41 patients in one clinical survey) (8, 11).

A direct link between *GLDC* loss of function and hydrocephalus was confirmed by analysis of mice carrying hypomorphic (*Gldc^GT1^*) or null (*Gldc^GT2^*) alleles of the murine homolog, *Gldc*. A proportion of *Gldc* mutants die perinatally, owing to neural tube defects (NTDs) resulting from failed neural tube closure (12, 13). However, among *Gldc-*deficient mice that survive postnatally, hydrocephalus becomes evident by 5–7 weeks of age in 20%–25% of homozygotes (*Gldc^GT1/GT1^*), with a characteristic domed head, distorted cranium, and severely enlarged lateral ventricles (12). These mice also show signs of NKH, including elevated glycine concentration in body fluids and tissues and premature lethality (12).

A prenatal origin of hydrocephalus was demonstrated by histological analysis of litters at embryonic day 18.5 (E18.5), which revealed enlarged lateral ventricles in *Gldc-*deficient (*Gldc^GT1/GT1^* or *Gldc^GT1/GT2^*) mice (12, 14). This is consistent with the association of ventriculomegaly, enlargement of the cerebral ventricles, with postnatal hydrocephalus in humans (15). Serial imaging of individual *Gldc^GT1/GT2^* fetuses at successive gestational time points by in utero high-frequency ultrasound showed onset of ventriculomegaly between E16.5 and E18.5 (14).

The physiological (communicating vs. noncommunicating) and metabolic mechanisms underlying *GLDC-*related hydrocephalus have not been determined. The GCS is a mitochondrial enzyme complex that mediates decarboxylation of glycine, with concomitant transfer of a 1-carbon group to tetrahydrofolate (THF), generating 5,10-methylene THF in folate 1-carbon metabolism (FOCM) (9). Hence, GCS loss of function not only causes accumulation of glycine, but also prevents transfer of glycine-derived 1-carbon units to FOCM (13). Excess glycine is thought to lead to neurological features of NKH, such as epilepsy (8, 9), whereas NTDs result from impaired FOCM (13). Potential mechanisms underlying the development of ventriculomegaly and hydrocephalus could therefore include consequences of FOCM suppression or glycine accumulation in CSF or neural tissues. For example, it is proposed that altered osmolality of CSF (a potential effect of excess glycine) could result in net movement of water into the ventricles, leading to abnormal CSF hydrodynamics (16). Alternatively, FOCM is required for provision of 1-carbon groups for key cellular processes including nucleotide biosynthesis and methylation reactions, whose disturbance could plausibly lead to ventriculomegaly. Here, we investigated the cause of hydrocephalus and the requirement for FOCM in ventricular development in Gldc-deficient mice.

## Results and Discussion

**Figure 1:**
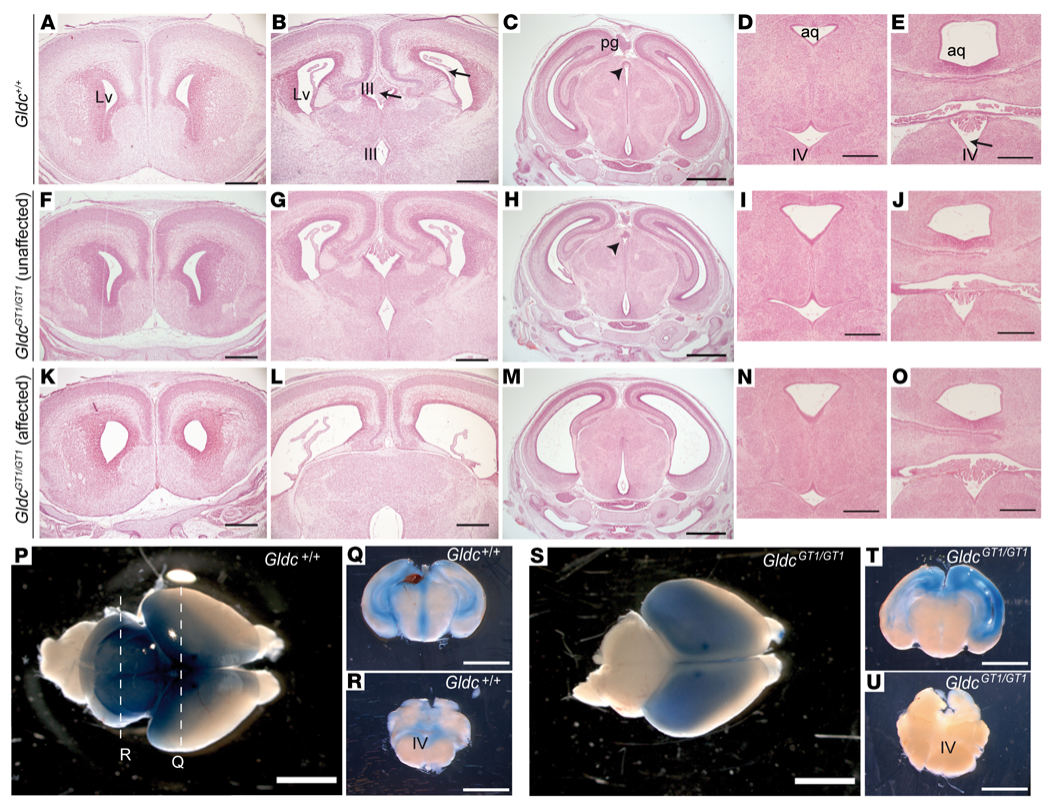
*Gldc* deficiency results in ventriculomegaly. Unlike wild-type (**A**–**E**) and unaffected *Gldc^GT1/GT1^* (**F**–**J**) fetuses, a subset (7 of 13) of *Gldc^GT1/GT1^* fetuses (**K**–**O**) were affected by enlargement of the lateral (Lv) and third (III) ventricles at E18.5. The fourth ventricle (IV, arrow in **E**) does not differ in size between genotypes (compare **D** with **E**, **I** with **J**, and **N** with **O**), nor does the aqueduct (aq) at this posterior axial level. Choroid plexus is detected in lateral, third, and fourth ventricles (arrows in **B**) of all genotypes. However, the pineal gland (pg) and subcommissural organ (arrowheads in **C** and **H**) are absent in *Gldc^GT1/GT1^* fetuses displaying ventriculomegaly (**M**). (**P**–**U**) Following bilateral injection into the lateral ventricles of neonatal mice (**P**, **Q**, **S**, and **T**), dye distributed throughout the ventricular system including the fourth ventricle (IV) of *Gldc*^+/+^ (**R**) but not *Gldc*^GT1/GT1^ (**U**) mice. Scale bars: 1 mm (**C**, **H**, and **M**), 5 mm (**P**–**U**), and 0.5 mm (other panels).

In our previous ultrasound analysis of litters at E18.5, *Gldc-*deficient (*Gldc^GT1/GT2^*) fetuses that exhibited enlarged lateral ventricles did not differ from wild types in volume of the fourth ventricle or cerebellum (14). Similarly, we found that among *Gldc^GT1/GT1^* fetuses with obvious dilatation of the lateral and third ventricles (7 of 13 *Gldc^GT1/GT1^*), more posterior structures including the fourth ventricle appeared comparable to wild types (Figure 1).

**Figure 2:**
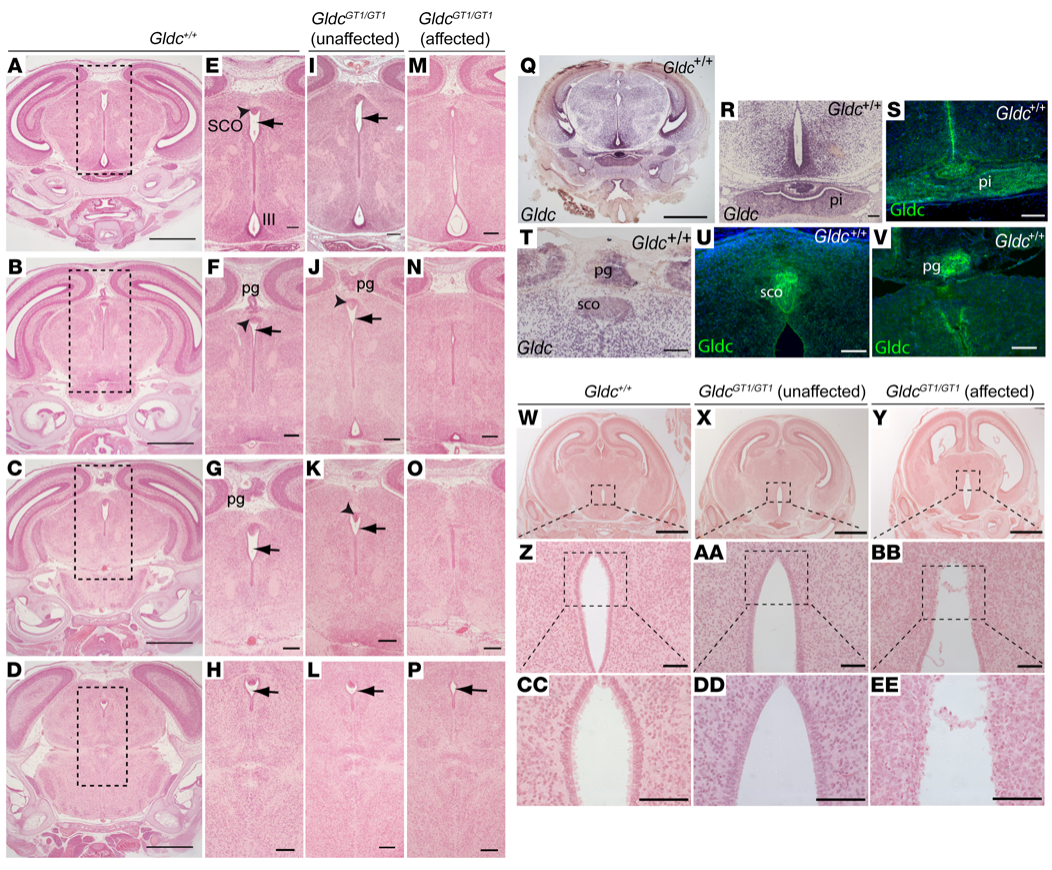
Ventriculomegaly is associated with aqueduct stenosis in *Gldc-*deficient fetuses. Coronal sections in a rostral-caudal sequence (at levels shown in **A**–**D** in wild-type brain) show continuity of the aqueduct of Sylvius in *Gldc^+/+^* (arrows in **E**–**H**) and unaffected *Gldc^GT1/GT1^* (**I**–**L**) fetuses at E18.5. In contrast, the aqueduct narrows and exhibits discontinuities in *Gldc^GT1/GT1^* mutants with ventriculomegaly (affected) (**M**–**P**). Boxed areas in **A**–**D** show enlarged regions in **E**–**P**. At E16.5, *Gldc* mRNA is widely expressed in the brain (**Q**, **R**, and **T**), with abundant expression in the pineal gland (pg), subcommissural organ (sco), and pituitary (pi). Immunohistochemistry confirms localization of Gldc protein at these sites at E18.5 (**S**, **U**, and **V**). The ependymal cell lining of the third ventricle (boxed in **W**–**Y**, enlarged in **Z**–**EE**) appears disrupted in *Gldc^GT1/GT1^* fetuses at E18.5 (**Y**). Scale bars: 0.1 mm (**R**–**V** and **Z**–**EE**), 0.5 mm (**A**–**P**), and 1 mm (**Q** and **W**–**Y**).

Specific expansion of the ventricular system rostral to the aqueduct of Silvius indicated the possibility of mechanical obstruction. Consistent with this hypothesis, after injection into the lateral ventricles, aqueous dye circulated to the posterior aqueduct and fourth ventricles in wild-type and *Gldc*^GT1/+^ neonatal mice but not in some *Gldc^GT1/GT1^* littermates (Figure 1, P–U and Supplemental Figure 1; supplemental material available online with this article; https://doi.org/10.1172/JCI132360DS1). Among wild-type and unaffected *Gldc^GT1/GT1^* fetuses at E18.5, the aqueduct lumen was continuous and could be followed in serial sections from the third to fourth ventricles (Figure 2). In contrast, the aqueduct was very narrow or completely occluded in all *Gldc^GT1/GT1^* fetuses that displayed ventriculomegaly (Figure 2, M–P, and Supplemental Figure 2).

At E18.5, the subcommissural organ (SCO) and pineal gland are visible in the roof of the aqueduct in wild-type and unaffected *Gldc*-deficient fetuses (Figure 2, A–L). However, in all the affected *Gldc*-deficient fetuses the SCO was absent and the pineal gland was absent or malformed (Figure 1M and Figure 2, M–P). SCO agenesis is associated with development of both communicating and noncommunicating forms of hydrocephalus in mice, as seen in genetic mutants for *Msx1*, *Pax6*, and *Rfx3* or overexpression of *Sox3* (17, 18). A hypoplastic pineal gland is also observed with postnatal hydrocephalus in *Lhx9*-null mice (19).

*Gldc* is expressed throughout the neuroepithelium at E9.5 (12). At developmental stages when ventriculomegaly arose, we found widespread expression of *Gldc* in the brain at E16.5, including the pineal gland, SCO, and pituitary gland (Figure 2, Q, T, and R). Immunostaining confirmed the presence of Gldc protein in these structures at E18.5 (Figure 2, S, U, and V).

Hydrocephalus has been associated with denudation of the ependymal cell layer lining the ventricles and subsequent occlusion of the aqueduct (17, 20). Lack or abnormal function of ependymal motile cilia may also result in hydrocephalus without aqueduct stenosis, possibly owing to impaired CSF flow (17, 20). We found that the ependymal cell layer was intact in the ventricular system of wild-type (*n* = 5) and unaffected *Gldc^GT1/GT1^* (*n* = 4) fetuses at E18.5 (Figure 2, W, X, Z, AA, CC, and DD, and Supplemental Figure 2). Abnormalities were not observed in the ependymal cell layer of the lateral ventricles in *Gldc^GT1/GT1^* fetuses with ventriculomegaly (Supplemental Figure 2, G–L), but this layer appeared disrupted in the dorsal region of the third ventricle in most (4 out of 5; Figure 2, Y, BB, and EE) but not all (Supplemental Figure 3) fetuses. The fetal onset of ventriculomegaly, prior to maturation of multiciliated ependyma (in the first postnatal week in mice), and largely intact ependyma suggest that disruption of this cell layer is unlikely to be causal. Together, the presence of enlarged lateral and third ventricles, unaffected fourth ventricle, impaired dye distribution, and late-fetal onset of ventriculomegaly implicate aqueduct stenosis as the structural abnormality causing a noncommunicating form of hydrocephalus in Gldc-deficient fetuses.

We next investigated the metabolic basis of hydrocephalus in *Gldc*-deficient mice. Loss of function of GCS causes accumulation of glycine, with significantly elevated levels in *Gldc*-deficient embryos by E11.5 (12). Metabolic labeling shows that the contribution of glycine-derived 1-carbon units to FOCM is also ablated in *Gldc*-deficient embryos, with consequent alterations in the relative abundance of folates (12, 13).

**Figure 3:**
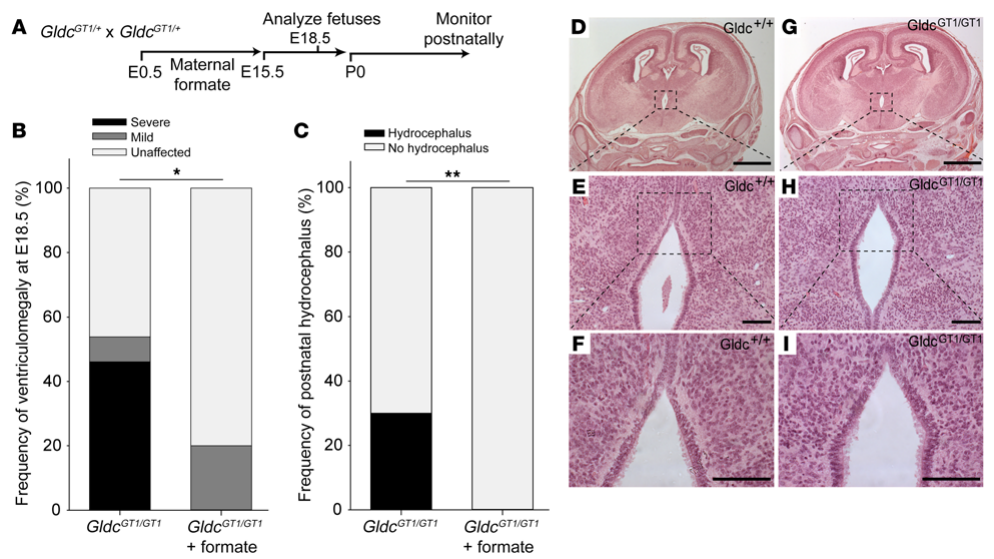
Maternal formate supplementation prevents ventriculomegaly and hydrocephalus. (**A**) *Gldc^GT1/+^* mice were mated and pregnant females were supplemented with formate from E0.5 to E15.5. (**B**) At E18.5, there was a significantly greater proportion of *Gldc^GT1/GT1^* fetuses that were unaffected (*n* = 8 of 10) in formate-treated litters than among *Gldc^GT1/GT1^* fetuses (*n* = 6 of 13) in control litters. *P < 0.05 by Fisher’s exact test. (**C**) Among offspring of formate-supplemented mice that were monitored at postnatal stages, none of the *Gldc^GT1/GT1^* mice (*n* = 0 of 35) developed hydrocephalus, whereas 30% (6 of 20) of nonsupplemented *Gldc^GT1/GT1^* offspring developed hydrocephalus by 6–7 weeks. **P < 0.002 by Fisher’s exact test. Number of litters: *n* = 19 supplemented and 35 nonsupplemented. (**D**–**I**) The ependymal lining of the third ventricle appeared intact in formate-treated fetuses at E18.5. Scale bars: 1 mm (**D** and **G**) and 100 μm (other panels).

We found that exogenous supply of 1-carbon units by maternal formate supplementation normalized the folate profile in *Gldc*-deficient embryos and prevented NTDs, despite the tissue glycine concentration remaining elevated (12, 13). Based on this strategy, pregnant dams were supplemented with formate prior to collection of litters for evaluation of ventriculomegaly (Figure 3A). Histological examination divided *Gldc^GT1/GT1^* fetuses into 2 main categories: a group that exhibited severely dilated lateral ventricles with absent SCO and pineal gland (Figure 1M) and another with normal appearance, indistinguishable from wild type (Figure 1H and Supplemental Figure 4). A few fetuses displayed an intermediate phenotype in which the SCO or pineal gland was detectable but appeared abnormal, accompanied by mild ventricle dilation (Supplemental Figure 4). Formate supplementation led to significant normalization of development (Figure 3B), with none of the treated *Gldc^GT1/GT1^* fetuses displaying severe ventriculomegaly or absence of the SCO and pineal gland. Similarly, the ependymal cell layer was intact in all formate-treated fetuses examined (*n* = 6 unaffected and 1 intermediate; Figure 3, D–I).

Having found that formate supplementation could prevent fetal ventriculomegaly, we asked whether this prenatal treatment rescued postnatal onset of hydrocephalus. *Gldc^GT1/+^* dams were supplemented with formate for the first 15 days of pregnancy and offspring were monitored for development of abnormalities until 6–7 weeks of age. Notably, none of the *Gldc^GT1/GT1^* offspring (*n* = 35) of formate-supplemented mice developed hydrocephalus, unlike nonsupplemented *Gldc^GT1/GT1^* in which hydrocephalus arose in 30% of mice (Figure 3C), similar to previous studies (12, 14).

**Figure 4:**
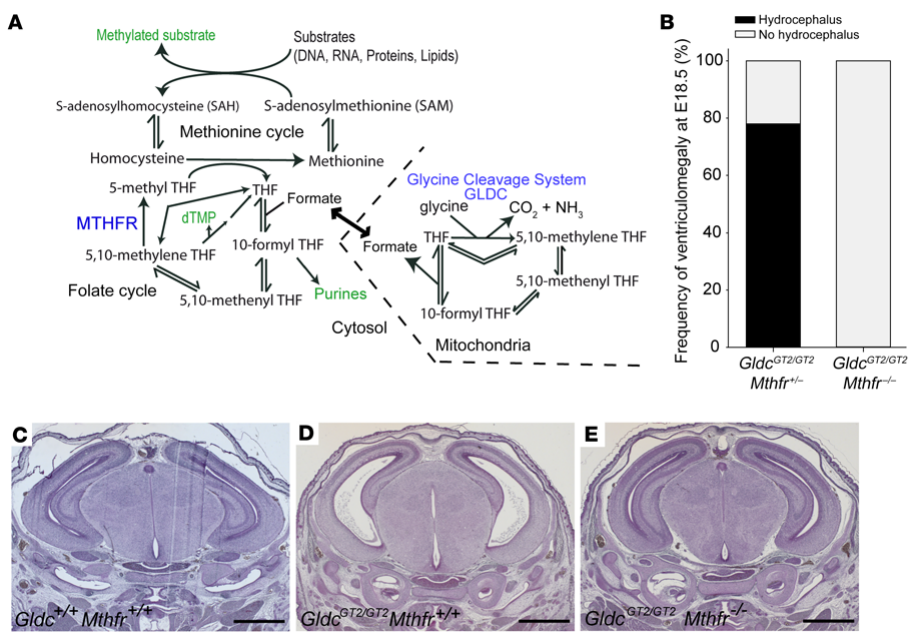
Mthfr deletion in *Gldc*-deficient mice normalizes development of the aqueduct, subcommisural organ, and pineal gland. (**A**) Outline of folate and methionine cycles, with key outputs in green text and relevant enzymes in blue text. Lack of Mthfr activity prevents transfer of 1-carbon units from 5,10-methylene THF to the methionine cycle via 5-methyl THF. (**B**) Among offspring of *Gldc^GT2/+^* *Mthfr^+/–^* intercrosses, ventriculomegaly was not detected at E18.5 in *Gldc^GT2/GT2^* *Mthfr^–/–^* fetuses (*n* = 5), but occurred at high frequency among *Gldc^GT2/GT2^* fetuses that were heterozygous (*n* = 7) or wild type (*n* =2) for *Mthfr* (*P* < 0.025 by Fisher’s exact test). (**C**–**E**) Sections of E15.5 wild-type (**C**), *Gldc^GT2/GT2^* (**D**), and *Gldc^GT2/GT2^ Mthfr^–/–^* (**E**) brains showing that absence of Mthfr restores the wild-type appearance of the pineal gland, subcommissural organ, and aqueduct, and prevents ventriculomegaly, in *Gldc*-null fetuses. Scale bars: 1 mm.

Prevention of ventriculomegaly/hydrocephalus by prenatal formate supplementation implicates insufficient supply of 1-carbon groups to FOCM as the underlying causative mechanism. The folate cycle intermediates, 5,10-methylene tetrahydrofolate (THF) and 10-formyl THF, act as 1-carbon donors in biosynthesis of thymidylate and purines. Alternatively, 1-carbon groups can be transferred to the methionine cycle via donation of a methyl group from 5-methyl THF to homocysteine (Figure 4A). To ask whether ventriculomegaly derives from suppression of the folate cycle or inadequate supply of 1-carbon groups to the methionine cycle we bred *Gldc-*deficient fetuses that lack 5,10-methylene tetrahydrofolate reductase (Mthfr). Compound null *Gldc^GT2/GT2^* *Mthfr^–/–^* embryos cannot generate 5-methyl THF and 1-carbon groups are therefore retained in the folate cycle (13).

Analysis of litters produced by intercross of compound heterozygous, *Gldc^GT2/+^* *Mthfr^+/–^* mice revealed venticulomegaly in *Gldc^GT2/GT2^* fetuses that were wild type or heterozygous at the *Mthfr* locus (Figure 4). In contrast, all *Gldc*/*Mthfr* double-knockout fetuses lacked ventriculomegaly, demonstrating a significant protective effect of the *Mthfr* null mutation. This preventive effect highlights the folate cycle, rather than methionine cycle, as the subset of FOCM reactions that are critical in prevention of aqueduct stenosis. Given the key role of the folate cycle in providing precursors for thymidylate and purine synthesis we speculate that defects result from impaired nucleotide biosynthesis in the neuroepithelium. The next step in this research will be to further refine the developmental stage and tissue site(s) in which folate cycle disruption subsequently leads to malformations of the SCO, pineal gland, and aqueduct.

In animal models, a potential link between folate status and hydrocephalus was noted in dietary studies examining nutritional effects of folate and vitamin B_12_ (21, 22). Some neonatal offspring of rats fed synthetic diets lacking folic acid and vitamin B_12_ exhibited enlarged ventricles attributed to possible blockage of the cerebral aqueduct (21). However, speculation remained over the relative importance of folic acid and vitamin B_12_ and whether FOCM was impaired in these models (22).

Abnormal folate transport to the brain has been proposed to contribute to aqueduct obstruction in the hydrocephalic Texas (H-Tx) rat (23, 24). Interestingly, however, hydrocephalus is not commonly described among neurological signs associated with the very low levels of folate in CSF that occur in hereditary folate malabsorption, caused by mutations in *SLC46A1* (proton-coupled folate transporter; PCFT) or cerebral folate deficiency, caused by mutations in *FOLR1* (folate receptor α; FRα) (25–27). On the other hand, hydrocephalus can arise in patients with severe MTHFR deficiency or remethylation disorders (including cblC disease) (28). MTHFR-related hydrocephalus may be of the communicating form (29), and it is not clear to what extent aqueduct stenosis also contributes. Nevertheless, impaired methionine synthase activity (as in cblC patients) could lead to accumulation of 5-methyl THF and thereby deplete 1-carbon units from the folate cycle (methyl trap). This suggests the potential for a shared biochemical mechanism underlying hydrocephalus caused by methionine synthase or Gldc deficiency.

Here, we found that impaired enzymatic activity within mitochondrial FOCM, as opposed to diminished exogenous supply or a methyl trap, can be a direct cause of aqueduct stenosis, the most common known cause of congenital hydrocephalus in humans. The presence of hydrocephalus and/or enlarged ventricles in NKH patients and in *Gldc-*deficient mice suggests that the requirements for GCS activity are shared. Mutations in *GLDC* may also predispose to failed neurulation in humans as in mice, missense variants having been identified in some patients with NTDs (30–32), including functional mutations found in both NTDs and NKH (30, 31).

Further evidence for a molecular link between congenital hydrocephalus and NTDs has come from identification of putative causal mutations in *TRIM71*, *SMARCC1*, and *PTCH1* in patients with communicating and obstructive forms of hydrocephalus (6). Loss of function of each of these genes in mice causes cranial NTDs (33–35). Although the association of hydrocephalus with spina bifida is usually considered a secondary manifestation, one could speculate that they may be independent malformations with a shared genetic origin in some individuals. Hence, although NTDs and hydrocephalus arise at different developmental stages, the potential for prevention of both defects by formate supplementation suggests that there may be a related cellular mechanism, serving as a starting point for further research toward understanding the causation, pathogenesis, and primary prevention of these related conditions.

## Methods

Further details are provided in the supplemental material.

*Gldc*-deficient mice carried gene-trap alleles denoted *Gldc^GT1^* (12) or *Gldc^GT2^* (13). *Mthfr*-null mice were previously described (36). Litters were generated by overnight matings, with the following day designated E0.5. Injection of the lateral ventricles was performed at P1. Mice were genotyped by PCR of genomic DNA (12, 13). Sodium formate (30 mg/mL) was added to drinking water (12, 13) from E0.5 to E15.5 and dams were then returned to normal drinking water.

Bouin’s fixed, paraffin-embedded samples were sectioned (8 μm) and stained with hematoxylin and eosin. In situ hybridization was performed on sections using a digoxygenin-labeled antisense probe for *Gldc* (12). Immunostaining was performed using anti-Gldc (1:300; Atlas Antibodies, HPA002318) with Invitrogen anti-rabbit Alexa Fluor secondary (Thermo Fisher Scientific, A11034) antibodies.

### Study approval.

Studies were carried out under regulations of the Animals (Scientific Procedures) Act 1986 of the United Kingdom (UK) Government and approved by the UCL Animal Welfare and Ethical Review Body, London, UK.

### Statistics.

Statistical analysis was performed by Fisher’s exact test, with *P* less than 0.05 considered significant.

## Author contributions

NDEG, AJC, and KYL designed the study. CS, YJP, MRM, KYL, DS, and SNW conducted the investigations. NDEG wrote the manuscript, which was edited by NDEG and AJC.

## Supplementary Material

Supplemental data

## Version 1 12/03/2019

In-Press Preview

## Version 2 02/04/2020

Electronic publication

## Version 3 03/02/2020

Print issue publication
