# Supplementary material for: Impaired folate 1-carbon metabolism causes formate-preventable hydrocephalus in glycine decarboxylase–deficient mice
Source: J Clin Invest. 2020 Feb 4;130(3):1446–52. doi: 10.1172/JCI132360 (PMC7269562; doi:10.1172/JCI132360)
Supplement: Supplemental data [file jci-130-132360-s142.pdf]

Supplementary Material

Methods

Mice and study approval

Gldc-deficient mice carried gene-trap alleles denoted $Gldc^{GT1}$ (1) or $Gldc^{GT2}$ (2) on a C57BL/6 background. *Mthfr* null mice were previously described (3). *Mthfr/Gldc* interaction studies were performed on a principally $Gldc^{GT2}$ strain (but not fully isogenic) genetic background.

Mice were used for experimental matings from six weeks of age. Mice were maintained on a standard breeder diet (Teklad). Litters were generated by overnight matings and the day of finding a copulation plug was designated embryonic day 0.5 (E0.5). Animal studies were carried out under regulations of the Animals (Scientific Procedures) Act 1986 of the UK Government, and in accordance with the guidance issued by the Medical Research Council, UK in *Responsibility in the Use of Animals for Medical Research* (July 1993).

Histology

Fetuses were fixed overnight in Bouin's solution (Sigma), dehydrated through an ethanol series, embedded in paraffin-wax, sectioned at 8 µm thickness and stained with haematoxylin and eosin.

Injection of the lateral ventricles

Pups at post-natal day 1 (P1; n = 16 pups from 4 litters) from 4 were chilled on ice and injected bilaterally with 2 µl of 0.4% trypan blue into each ventricles at a position 2/5 of the distance between the eye and Lambda as described (4). Pups were culled and the brain fixed in 4% paraformaldehyde and photographed prior to analysis of 1-2 mm tissue slices or 200 µm vibrotome sections.

Expression analysis

For mRNA and protein expression analysis, embryos were fixed in 10% formalin and dehydrated through an ethanol series prior to wax embedding and sectioning. *In situ* hybridisation was performed on sections using a digoxygenin-labelled anti-sense probe to *Gldc* (1). Immunostaining was performed using anti-Gldc (1:300; Atlas Antibodies, HPA002318) with anti-rabbit AlexaFluor secondary (Invitrogen Thermo Fisher, A11034) antibodies and DAPI staining of nuclei. Images were captured using Axiovision v4.8.2 (Zeiss) or µManager v1.4 (Open Imaging) software.

Statistical analysis was performed by Fisher-Exact test with $p<0.05$ considered significant, using Sigmastat (v3.5, Systat Software).

References

1. Pai,Y.J., Leung,K.Y., Savery,D., Hutchin,T., Prunty,H., Heales,S., Brosnan,M.E., Brosnan,J.T., Copp,A.J., and Greene,N.D. 2015. Glycine decarboxylase deficiency causes neural tube defects and features of non-ketotic hyperglycinemia in mice. *Nat. Commun.* **6**:6388.

2. Leung,K.Y., Pai,Y.J., Chen,Q., Santos,C., Calvani,E., Sudiwala,S., Savery,D., Ralser,M., Gross,S.S., Copp,A.J. et al 2017. Partitioning of One-Carbon Units in Folate and Methionine Metabolism Is Essential for Neural Tube Closure. *Cell Rep.* **21**:1795-1808.

3. Chen,Z., Karaplis,A.C., Ackerman,S.L., Pogribny,I.P., Melnyk,S., Lussier-Cacan,S., Chen,M.F., Pai,A., John,S.W., Smith,R.S. et al 2001. Mice deficient in methylenetetrahydrofolate reductase exhibit hyperhomocysteinemia and decreased methylation capacity, with neuropathology and aortic lipid deposition. *Hum. Mol. Genet.* **10**:433-443.

4. Kim,J.Y., Ash,R.T., Ceballos-Diaz,C., Levites,Y., Golde,T.E., Smirnakis,S.M., and Jankowsky,J.L. 2013. Viral transduction of the neonatal brain delivers controllable genetic mosaicism for visualising and manipulating neuronal circuits in vivo. *Eur. J. Neurosci.* **37**:1203-1220.

Supplementary Figures

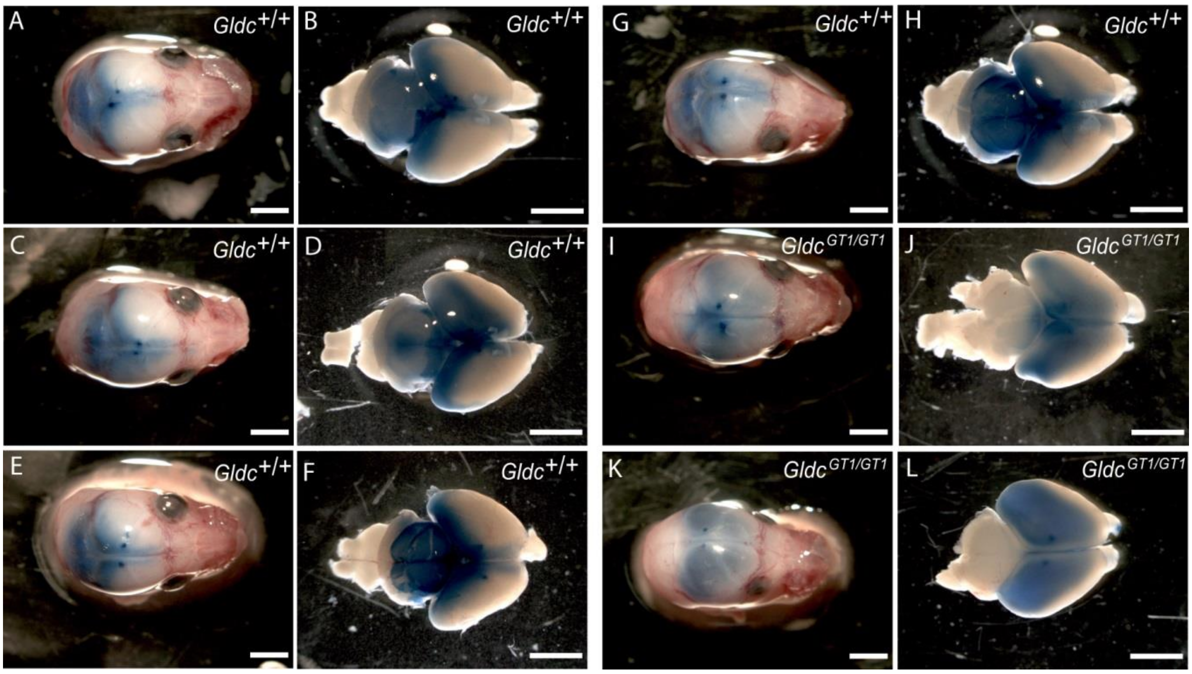

Figure S1. Dye injection into the lateral ventricles of wild-type and *Gldc*-deficient mice. *Gldc*$^{+/+}$ and *Gldc*$^{GT1/GT1}$ mice were injected with 0.4% trypan blue at post-natal day 1. The injection sites are visible in each pup (after removal of skin). After removal of the skull the distribution of dye in the brain is visible (paired images A-B, C-D, E-F, G-H, I-J, K-L are corresponding samples). Dye distribution encompassed the posterior region, including the fourth ventricle, in *Gldc*$^{+/+}$ mice but not in *Gldc*-deficient pups with ventriculomegaly. Scale bars represent 5 mm.

The *Gldc*$^{+/+}$ panel in Figure 1C is reshown in Figure S1H. The *Gldc*$^{GT1/GT1}$ panel in Figure 1S is reshown in Figure S1L.

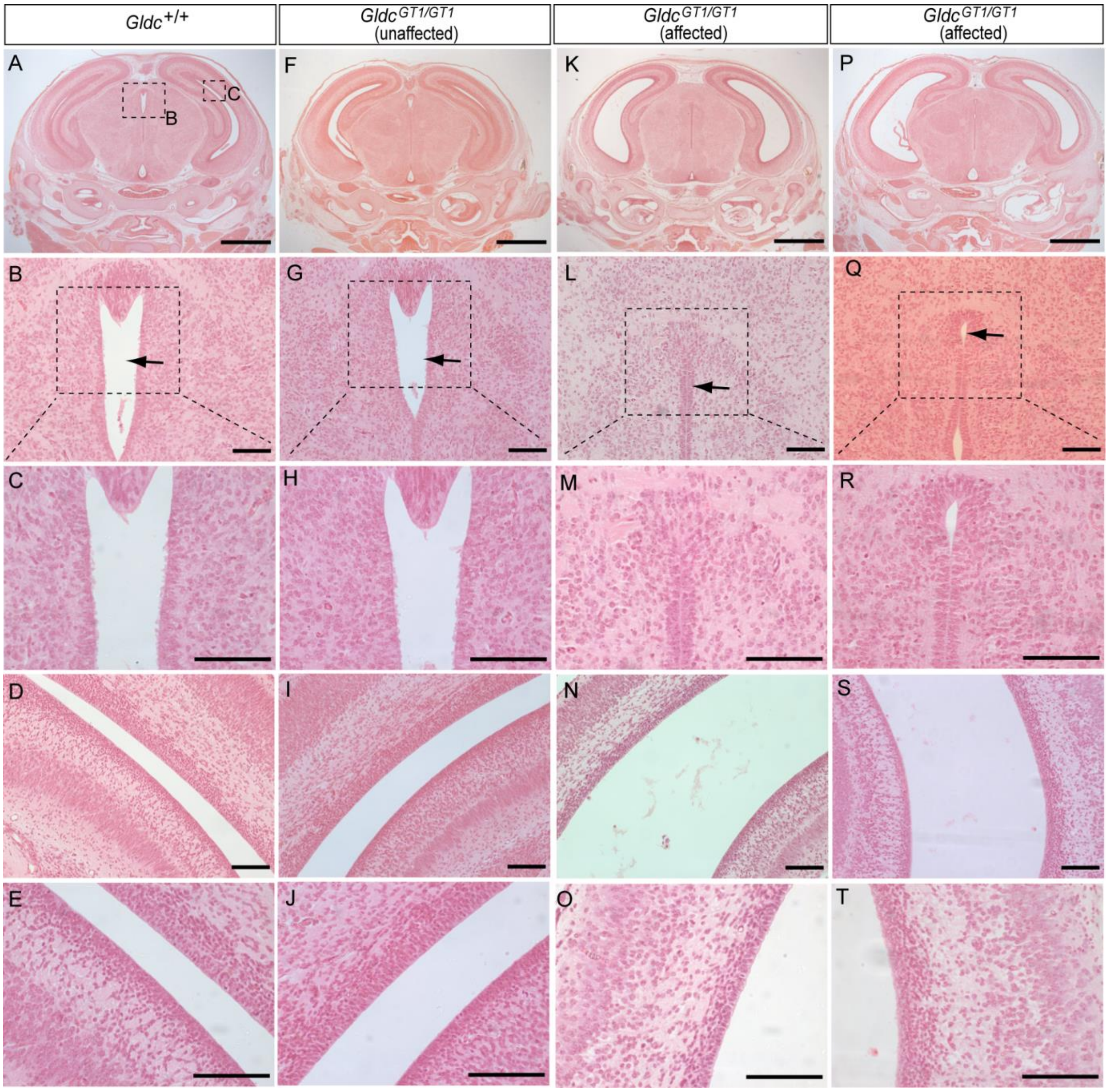

Figure S2. Ependymal cell lining is intact in the lateral ventricles and aqueduct of wild-type and *Gldc*-deficient foetuses at E18.5. Representative images of haematoxylin and eosin stained sections of *Gldc*$^{+/+}$ (A-E'), unaffected *Gldc*$^{GT1/GT1}$ (F-J) and *Gldc*$^{GT1/GT1}$ fetuses with ventriculomegaly (K-T). Where visible, the lining of the aqueduct (B, G and enlarged in C, H) was intact, although the aqueduct was occluded or very small in *Gldc* mutants with ventriculomegaly (L-M, Q-R). The appearance of the lining of the lateral ventricles did not differ between genotypes (D, I, N, S and enlarged in E, J, O, T) irrespective of ventriculomegaly. Scale represents 1 mm in A, F, K, P and 100 µm in all other panels.

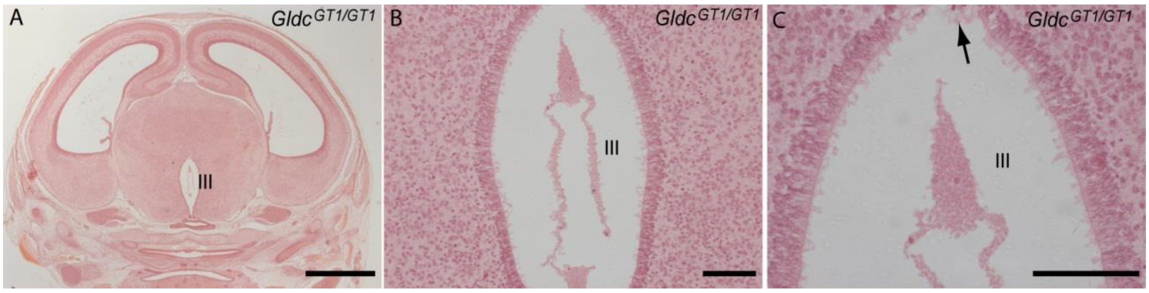

Figure S3. Ependymal cell layer is intact in the in third ventricle of a *Gldc*$^{GT1/GT1}$ mutant with ventriculomegaly and aqueduct stenosis at E18.5. In contrast to other *Gldc*$^{GT1/GT1}$ mutants (4 of 5), sections through the third ventricle (III) of 1 mutant showed that the ependymal cell layer was intact, with possible exception of a small region at the dorsal aspect (arrow in C). *S*ections of the lateral ventricle and aqueduct of the same fetus are shown in Supplementary Fig. 2G-I. Scale bar represents 1 mm in A and 100 µm in B-C.

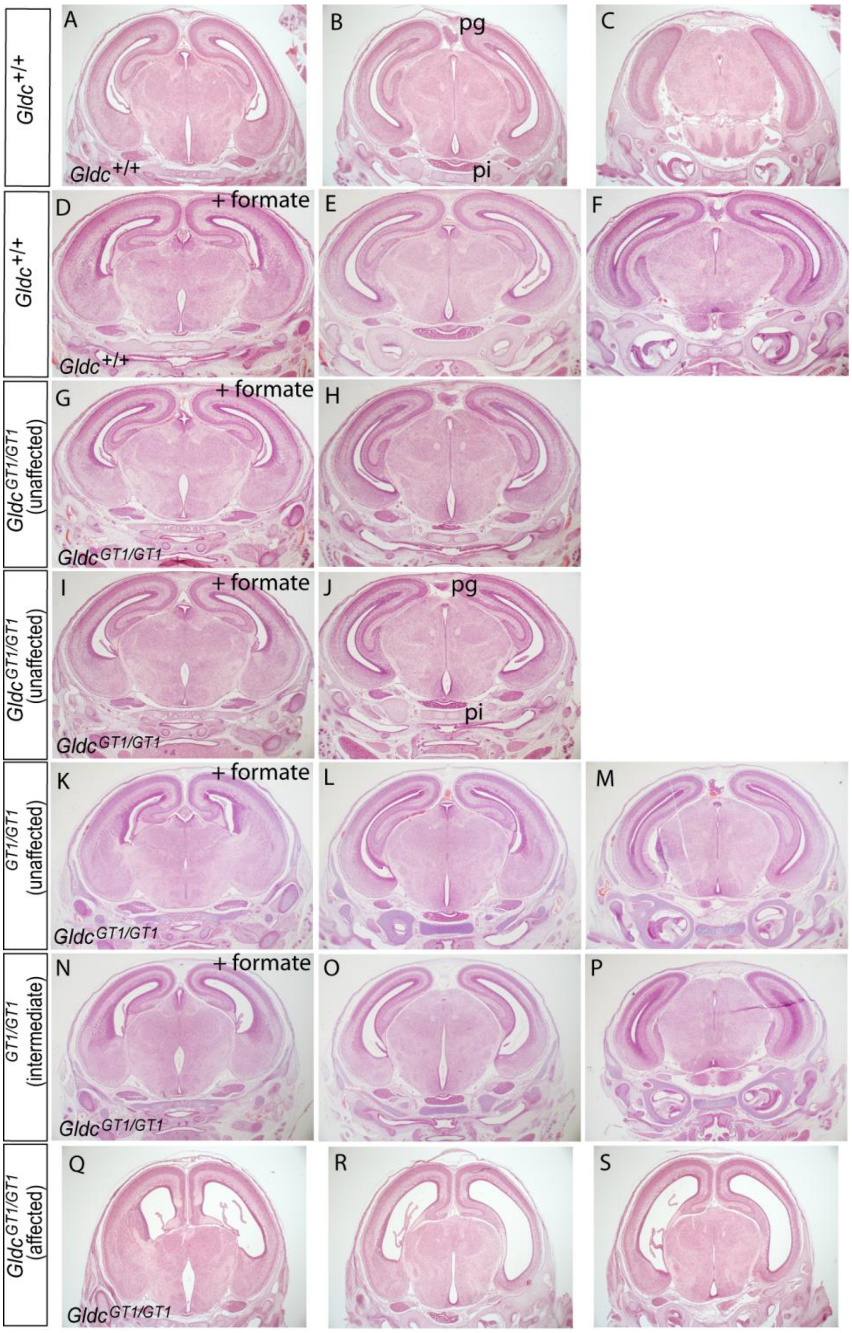

Figure S4. Phenotypes among *Gldc*-deficient fetuses at E18.5 (untreated or exposed to maternal formate supplementation). Typical appearance of coronal sections through the lateral ventricles around the level of the pineal gland (pi). Sections are in anterior to posterior sequence within samples (e.g. A-C) and matched for axial level in columns (e.g. A, D, G, I, K, N, Q). The typical appearance of unaffected *Gldc*$^{GT1/GT1}$ fetuses (G-M) does not differ from wild-types (A-F), whereas affected foetuses show enlarged lateral ventricles and absent gland (Q-S). An intermediate phenotype, comprising moderately enlarged ventricles and absent or malformed pineal gland (N-P) was observed among a subset of *Gldc*$^{GT1/GT}$ foetuses.

The *Gldc*$^{+/+}$ panel in Figure 1C is reshown in Figure S4B.
